# Organisation of Wildlife Passive Disease Surveillance in Slovenia over 30 Years (1995–2025) and Insights into Certain Causes of Disease or Mortality

**DOI:** 10.3390/vetsci13040360

**Published:** 2026-04-07

**Authors:** Gorazd Vengušt, Diana Žele Vengušt

**Affiliations:** Veterinary Faculty, University of Ljubljana, Gerbičeva Ulica 60, 1000 Ljubljana, Slovenia

**Keywords:** wild animals health, monitoring, necropsies archival record, One Health

## Abstract

Wildlife health surveillance is a vital element of disease prevention, biodiversity conservation, and public health protection, especially as most emerging infectious diseases originate from wildlife. In Slovenia, long-term passive surveillance based on necropsy data has yielded valuable insights into wildlife mortality patterns over the past three decades, despite inherent limitations such as carcass detectability, reporting bias, scavenging, and decomposition. Ongoing cooperation among governmental institutions, veterinary services, hunters, and wildlife management organisations has enabled the effective operation of this system, although passive surveillance remains subject to spatial, temporal, and species-specific biases. Necropsy data show that infectious diseases, particularly parasitic infections, are the main causes of mortality in key species such as roe deer and chamois, reflecting both their population abundance and targeted monitoring. In contrast, carcasses of species such as wild boar, red deer, small mammals, and birds are underrepresented due to ecological factors, biosecurity constraints, or low detectability. Overall, while passive wildlife surveillance does not provide representative population-level mortality estimates, it remains a reliable tool for identifying the presence or absence of significant diseases and for understanding broad mortality patterns when interpreted in the context of known methodological and ecological limitations.

## Simple Summary

Many infectious diseases affecting humans and domestic animals originate from wildlife, making wildlife health surveillance essential for early disease detection as well as for biodiversity conservation. In Slovenia, long-term wildlife disease monitoring relies on post-mortem examinations of animals found dead and submitted by hunters and wildlife professionals. Although this passive surveillance approach is affected by carcass detectability, scavenger activity, and reporting bias, it provides valuable information on disease occurrence and major causes of mortality. The findings indicate that infectious diseases, particularly parasitic infections, are the leading causes of death in species such as roe deer and chamois. In contrast, small mammals, birds, and some large game species are underrepresented because their carcasses are difficult to detect or are not systematically examined. Overall, long-term necropsy-based surveillance remains an important tool for understanding wildlife health trends and supporting disease prevention, provided its limitations are carefully considered.

## 1. Introduction

Slovenia, a small country in Central Europe, is a biodiversity hotspot with a wide variety of ecosystems, plants, and animal species. The country’s diverse geographical features include the Julian Alps, the Dinaric Karst, the Pannonian Plain, and the Adriatic coast. These natural habitats support an impressive variety of flora and fauna, making Slovenia one of the most biodiverse countries in Europe. Almost 60% of Slovenia is covered by forests, which harbour a wide variety of native wild animal species, including rare and endangered species, and provide a home and shelter for the three largest carnivores: the brown bear, grey wolf, and Eurasian lynx [1,2].

Diseases in wildlife appear in numerous forms in a diverse group of animal species and populations around the globe and can pose a significant burden that impacts biodiversity conservation, wildlife management and the global economy. Wildlife can serve as an important reservoir for highly transmissible and virulent pathogens, many of which are also contagious to domestic animals or humans [3,4,5,6]. Roughly more than half of human infectious diseases are caused by pathogens shared with animals [7]. Health monitoring and surveillance are important tools for the detection and control of wildlife diseases and are also important components of the One Health concept [3,8,9,10]. The One Health framework promotes interdisciplinary collaboration among wildlife ecologists, veterinarians, and public health professionals, ensuring that disease surveillance addresses wildlife health and mitigates the risk of spillover to domestic animals and humans [6,11]. There is broad recognition that systematic surveillance of diseases in wild animal populations enhances national capacity to understand the epizootiology of infectious and zoonotic diseases and strengthens preparedness to protect wildlife, domestic animal, and human health [3]. Regular animal health surveillance provides essential epidemiological evidence of national disease-free status and confirms the absence of major infectious diseases in free-ranging wildlife species [12,13]. The surveillance and monitoring of wildlife diseases are long-term activities that should be implemented when appropriate legal frameworks exist. Early disease detection and sampling relies on both active and passive surveillance systems [14]. In general, once a pathogen’s occurrence and distribution are established, health surveillance continues with prevention, control, and, where applicable, eradication [15]. Surveys based on post-mortem examinations of animal carcasses can provide a comprehensive means of investigation of the cause of death and valuable information on the population health, including age and sex structure and causes of mortality [16,17]. In the context of passive surveillance of wildlife diseases, hunters are indispensable, providing samples and early notification of emerging diseases [18]. The involvement of hunters in the design and evaluation of surveillance and control measures, their motivation, trust and commitment have a direct impact on the acceptance and effectiveness of surveillance [19]. In addition to hunters, engagement with local communities, wildlife rangers, and citizen scientists contributes significantly to passive surveillance networks, increasing spatial coverage and enabling early detection of emerging diseases [20].

Wildlife disease analysis in Europe shows a strong focus on zoonoses and diseases shared with livestock, particularly in wild boar, deer, carnivores and birds [21,22,23]. Passive wildlife disease surveillance in Europe is a cornerstone of early warning for different diseases such as avian influenza, African and classical swine fever, West Nile virus, bovine tuberculosis, rabies, hepatitis E, tularemia, canine distemper, brucellosis, echinococcosis, and other high-impact diseases, especially where case fatality is high and resources are limited [21,22,23,24,25]. Surveillance capacity and methods are improving, but significant geographic, taxonomic and data-sharing gaps remain, reinforcing calls for harmonised, One Health-oriented, integrated wildlife monitoring [5,26,27]. In Europe, structured collaborations such as the European Wildlife Disease Association (EWDA) Network support the harmonisation of wildlife health surveillance (WHS) programmes, facilitate the exchange of expertise, and develop diagnostic and monitoring resources across countries. Although WHS is widely recognised as essential for detecting and managing disease risks in wild animal populations, the structure and extent of WHS programmes across Europe remain heterogeneous and incomplete [28]. A survey of European nations coordinated through the EWDA Network indicates that while some countries have implemented comprehensive or partial general wildlife health surveillance programmes, others primarily maintain disease-specific monitoring or rely on passive reporting (e.g., for rabies or classical swine fever), highlighting variability in national infrastructure and surveillance priorities [10,27,28].

This study provides an overview of 30 years (1995–2025) of continuous passive wildlife surveillance within a national health surveillance programme in Slovenia, which also serves as an indicator of successful cooperation between the Faculty of Veterinary Medicine, hunters, government, and the veterinary administration. Despite increasing recognition of the importance of wildlife disease surveillance within a One Health framework, there remains a notable lack of published studies in Europe using multi-species passive monitoring through post-mortem examinations. Most European countries instead focus their surveillance on single species or specific pathogens, rather than implementing integrated multi-species assessments [28,29].

## 2. Materials and Methods

Study area: Situated in Central Europe, Slovenia comprises four main geographic regions: the European Alps, the karstic Dinaric Alps, the Pannonian–Danubian lowlands and hills, and the Mediterranean coastline. Slovenia borders Italy to the west, Austria to the north, Hungary to the northeast, and Croatia to the south and southeast. The country also has a short coastline along the Adriatic Sea to the southwest, which forms part of the Mediterranean Sea. Most of Slovenia is mountainous and forested, covering about 60% of its total area of 20,273 km^2^.

According to the Institute of the Republic of Slovenia for Nature Conservation, 13% of the country’s territory is legally protected. The remaining 87%, excluding urban areas such as cities and industrial zones, consists of public and private land where hunting activities are regulated under state concessions. This distribution ensures that, while hunting is widespread, substantial areas of the country are reserved for wildlife conservation and monitoring.

**Figure 1:**
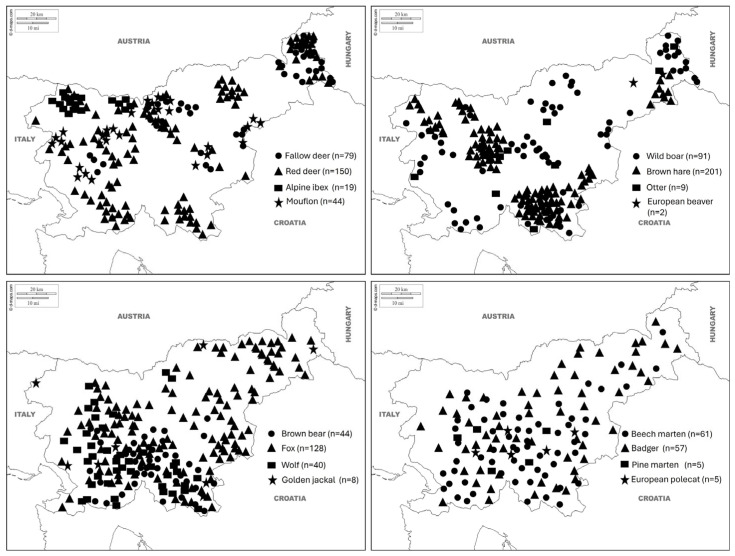
Sampling locations and numbers of various free-ranging wild mammal species collected over a 30-year period (1995–2025) through passive wildlife surveillance as part of a national health surveillance programme in Slovenia.

**Figure 2:**
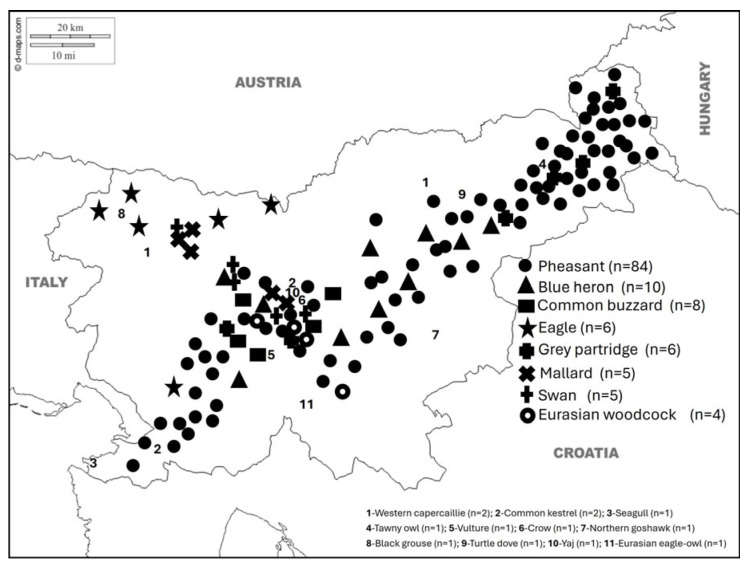
Sampling locations and numbers of various free-ranging avian species collected over a 30-year period (1995–2025) through passive wildlife surveillance as part of a national health surveillance programme in Slovenia.

Hunters from 411 hunting grounds managed by the Hunters Association of Slovenia and professional gamekeepers from eight special purpose state hunting grounds managed by the Slovenian Forest Service provided 2.516 fresh or frozen wild animal carcasses (mammal and avian) nationwide (Figure 1 and Figure 2). Locations of roe deer and chamois samples are published elsewhere [30,31].

Data: Carcasses of wild animals found dead, animals culled due to clinical signs of disease, or animals harvested during the regular annual cull that exhibited atypical health conditions during carcass processing were submitted for diagnostic examination to the Faculty of Veterinary Medicine at the University of Ljubljana. In all cases of diseased animals or corpse discovery, samples were collected randomly. Under state-issued concessions, hunters are permitted to harvest apparently healthy or diseased wildlife when the species falls within a legally defined hunting period, or when a special government decree allows the culling of protected species without a defined period. The concessions also authorise hunters to collect carcasses found in nature; carcasses of protected species must undergo examination at the Faculty of Veterinary Medicine, while other carcasses are evaluated by the hunting supervisor, who decides whether the carcass will be sent to the faculty or not. The removal of sick protected animals or individuals outside their designated hunting period requires authorisation from the hunting inspector. This framework ensures compliance with legal regulations while supporting wildlife health monitoring and management. The carcasses were systematically submitted through the Veterinary Hygiene Service (VHS), the competent authority responsible for the controlled collection and sanitary management of deceased animals in Slovenia. The data discussed in this paper are sourced from archival records of necropsies beginning in 1995, when our active involvement at the Institute commenced.

Comprehensive necropsies of all carcasses and tissues collected for histology were conducted throughout the study period according to protocols described by McAloose et al. [32]. Representative tissue and organ samples were collected for further analysis.

For bacteriological examination, tissue samples were typically cultured on blood agar containing 5% sheep blood. Isolates were initially characterized biochemically using the commercial API system (API bioMérieux, Lyon, France) and subsequently by matrix-assisted laser desorption ionization time-of-flight mass spectrometry (MALDI-TOF MS; Bruker Daltonik GmbH, Bremen, Germany). MALDI-TOF MS was introduced into routine microbiological diagnostics at the Faculty of Veterinary Medicine in 2015 and has since largely replaced biochemical identification.

For virological examination, the agar gel immunodiffusion assay, the microscopic agglutination test and the enzyme-linked immunosorbent assay were used to detect specific antibodies. Polymerase chain reaction (PCR) methods were used for the detection of viruses in blood and other tissues. When necessary, next-generation sequencing was used. Parasitological examinations were performed on fresh faecal samples and, where applicable, adult parasites collected from relevant organs. Faecal samples (approximately 10 g) were stored at 4 °C until analysis and examined using a flotation technique with a saturated sodium chloride solution and where relevant, sedimentation. Adult parasites were preserved in 70% ethanol and identified morphologically. DNA was also extracted from faecal or tissue samples for PCR using species-specific primers to confirm parasite identity. Although the full WAAVP (World Association for the Advancement of Veterinary Parasitology) guidelines were not strictly followed, all procedures were adapted from the recommendations by Soulsby [33], Taylor et al. [34], Eckert [35], Deplazes et al. [36], and Boch and Supperer [37] to ensure reproducibility and comparability of results.

## 3. Results

Over a 30-year period, 2370 mammalian and 146 avian necropsies were recorded, indicating a substantially higher representation of mammals than birds in the examined material. Necropsies were conducted on 22 mammal species, ranging from 989 necropsies of roe deer (*Capreolus capreolus*) to 2 of European beaver (*Castor fiber*). The four most frequently examined species were roe deer (*n* = 989), chamois (*Rupicapra rupicapra*) (*n* = 392), brown hare (*Lepus europaeus*) (*n* = 201), and red deer (*Cervus elaphus*) (*n* = 150), together accounting for approximately 66% of all mammal necropsies. Carnivores, mesopredators, and rare or elusive species, such as lynx (*Lynx lynx*) (*n* = 23), otter (*Lutra lutra*) (*n* = 9), and golden jackal (*Canis aureus*) (*n* = 8), were minimally represented. (Table 1). Avian necropsies were significantly fewer and showed a more uneven distribution among species. The most frequently examined bird was the ring-necked pheasant (*Phasianus colchicus*), with 84 necropsies, accounting for approximately 57.5% of all avian cases. The next most frequently examined avian species were the blue heron (*Ardea herodias*) (*n* = 10), and common buzzard (*Buteo buteo*) (*n* = 8) (Table 1). All other bird species were represented by fewer than ten necropsies each. Overall, the necropsy dataset was dominated by a limited number of common mammalian species, particularly cervids, while avian necropsies were both scarce and taxonomically fragmented. The distribution shows a pronounced imbalance in species representation, with a small subset of taxa accounting for most examined cases over the 30-year study period.

Analysis of the collected data revealed that mammal mortality in the studied area is primarily caused by a combination of anthropogenic factors and parasitic or infectious diseases, with clear species-specific patterns. Traffic accidents and firearms were the leading causes of death among large mammals, including red deer, wild boar, brown bears, wolves, and lynx. Small to medium-sized carnivores, such as foxes, beech martens, badgers, and European wildcats, also experienced high mortality due to traffic collisions and infectious diseases such as canine distemper. Parasitic infections played a major role in ungulate mortality, particularly in roe deer, chamois, Alpine ibex, and European mouflon, where multiple endoparasitism and *Sarcoptes scabiei* infestations contributed significantly to deaths. Multiple endoparasitism was present in all examined species of Slovenian wild ruminants, although prevalence varied between species (Table 1). Additionally, specific pathogens such as *Haemonchus contortus* in roe deer, and EBHS and MAP in brown hares were recorded. Medium-sized mammals, including otters, nutrias, European polecats, pine martens, and European beavers, also exhibited mortality linked to traffic accidents, with occasional contributions from dog predation or bacterial infections.

Birds were affected by both anthropogenic hazards and natural causes. Ground-nesting species, including pheasants and grey partridges, were primarily affected by trauma, firearms, and predation, while aquatic species such as mallards and blue herons were affected by traffic accidents, firearms, and cachexia. Raptors, including common buzzards and northern goshawks, were mainly affected by traffic accidents and firearms. Other observed causes in some species included sepsis and parasites. In some species, the advanced stage of decomposition prevented determination of the cause of death or disease.

**Figure 3:**
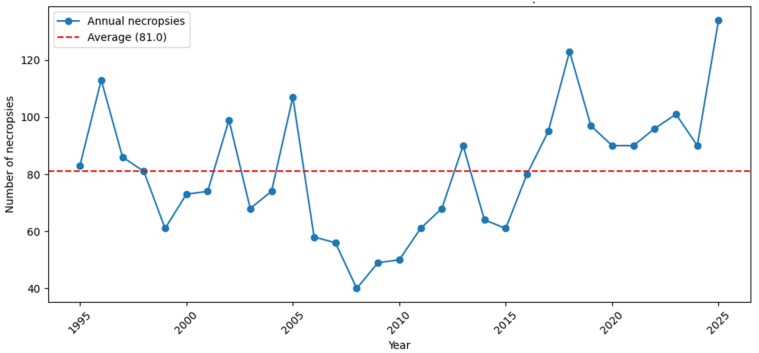
Number of post-mortem examinations of various free-ranging wild mammal and avian species at the Veterinary Faculty as part of a national health surveillance programme in Slovenia per year over 30 years (1995–2025).

Figure 3 presents annual data on wildlife post-mortem examinations conducted from 1995 to 2025. Over this period, the average was approximately 81 necropsies per year, although there was considerable year-to-year variation. Overall, the number of necropsies fluctuates markedly from year to year, with a noticeable increase in recent years. In the late 1990s and early 2000s, the annual number of examinations generally ranged from 60 to 110. A decline occurred between 2006 and 2010, reaching a minimum of 40 necropsies in 2008. The decline in the number of submitted carcasses is likely due to generational turnover at the faculty and organisational changes within the hunters’ association. After 2010, the numbers gradually increased, with a pronounced rise after 2016. The highest value was observed in 2025 (134 cases).

## 4. Discussion

Many infectious diseases originate from, or are carried by, wildlife and of those that have emerged in the past two decades, roughly 72% are estimated to have originated from wildlife [38,39,40]. Monitoring wildlife-associated diseases provides valuable insights into the causes of mortality and is necessary to identify new and re-emerging pathogens, while also ensuring awareness of the risks that these diseases may pose to wildlife, domestic animals, and human health [24,41]. Emerging diseases in wildlife also contribute to biodiversity loss, with inevitable direct and indirect effects on ecosystems [42,43]. For these reasons, wildlife health surveillance has become an essential part of national preparedness strategies in Europe [28,44], and this is also recognised in Slovenia. Long term necropsy-based programmes provide baseline health profiles and confirm the presence or absence of key pathogens, as demonstrated for roe deer and chamois in Slovenia [30,31]. In contrast to active disease surveillance, which is highly sensitive, focuses on a limited number of species or pathogens, and provides more complete and representative epidemiological data, passive disease surveillance covers a comprehensive range of wildlife and various causes of morbidity and mortality, spanning wide geographic areas and long time periods [5,10,45]. For example, studies of African swine fever in wild boar show that passive surveillance can detect early outbreaks, while active surveillance improves coverage and detection in low-density populations [14]. Although both passive and active surveillance strategies have been successfully applied in wildlife contexts, their implementation is often associated with unique challenges [46]. Importantly, passive surveillance is limited by carcass detection, reporting behaviour, scavenging, and decomposition, resulting in strong spatial, temporal, and demographic biases [47]. The establishment of the Department of Wildlife Healthcare and Breeding at the Veterinary Faculty (VF) in Ljubljana in 1953 marks the beginning of general wildlife health surveillance in Slovenia. Continuous good co-operation between numerous governmental and non-governmental institutions over decades enables the effective functioning of the disease surveillance system. Wildlife management in Slovenia is conducted by various state institutions. Protected animal species fall under the jurisdiction of the Ministry of Environment, Climate and Energy, while the Ministry of Agriculture, Forestry and Food (MAFF) is responsible for non-protected wild animal species. The latter also includes the Administration for Food Safety, Veterinary Sector and Plant Protection. All investigations involving wildlife are conducted at the Faculty of Veterinary Medicine, which includes the National Veterinary Institute (NVI) providing laboratory support. In Slovenia, the Veterinary Hygiene Service (VHS) is responsible for carcass collection and ensures their safe disposal. As a division of the NVI, the VHS plays a central role in preventing the spread of diseases to animals and humans by eliminating potential sources of infection. Additionally, their role is significant from an environmental perspective, as the disposal of dead animals prevents the pollution of water, soil, and animal feed. Hunting in Slovenia is organised within Special purpose state hunting grounds, where professional gamekeepers operate under the Slovenian Forest Service (SFS), established by the Republic of Slovenia to provide public forestry services in all Slovenian forests, regardless of ownership. In addition to state gamekeeping, the Hunters’ Association of Slovenia (HAS) is an independent non-governmental organisation dedicated to hunting and nature conservation. It operates in the public interest and represents a professional and unified association of hunters with a tradition spanning more than 110 years, making it one of the oldest hunting associations in Europe. The members of the HAS are organised into 411 hunting families with more than 20.000 members. There are also regional hunting organisations throughout Slovenia, as well as other societies and associations that promote the interests of hunting. All the organisations mentioned above are directly or indirectly involved in co-financing the disease monitoring system. Most of the funds are intended for active disease surveillance, i.e., for particularly dangerous diseases (e.g., influenza, rabies, *Echinococcus multilocularis*, *Trichinella* sp.) and economically important diseases (e.g., classical swine fever, African swine fever, Aujeszky’s disease), with a smaller share is also designated for passive surveillance.

We agree with Küker et al. [17] that data extracted from archived necropsy reports could serve as a useful resource for animal health surveillance and for providing an overview of disease incidence over a certain period in different animal species. For this article, we also used an archive from which we obtained data for the past three decades. During the 30-year period of passive wildlife surveillance in Slovenia, we conducted 2512 post-mortem surveys on various wild animals. Each year, between 40 and 134 wildlife carcasses have been submitted by hunters to our laboratory for analysis. Due to various factors affecting the submission of wildlife carcasses, those examined at necropsy are generally unrepresentative of the broader population and national wildlife distribution [48]. In our view, hunter-collected samples were likely neither systematic nor unbiased. They probably submitted for necropsy animals they found interesting—those with unusual signs or behaviours, potential trophy specimens, or carcasses that were still sufficiently preserved. Nevertheless, the absolute number of animals analysed was substantial, which is important because clinicopathological studies identify all disease processes affecting the examined animals [29,49]. We therefore consider this type of surveillance reliable for assessing the presence or absence of specific diseases and other causes of mortality in the population.

A few years ago, we conducted comprehensive twenty-year analyses of the causes of mortality in wildlife in Slovenia, focusing specifically on two species: roe deer and chamois. There is little information on specific diseases affecting roe deer, and studies of their overall health across Europe based on passive monitoring have only been conducted in France [50], Sweden [51], and Switzerland [41], while for chamois, the only comprehensive study was conducted in Slovenia [31]. Roe deer were subjected to necropsies more frequently, reflecting their abundance, as they are among the most widespread free-living ungulates in Slovenia, with an estimated population of 110,000 [52]. In roe deer, the main causes of mortality were infectious diseases (67%) and non-infectious conditions (28%). Parasitic infections accounted for 48% of deaths, bacterial infections for 14.8%, trauma for 12.5%, and metabolic disorders for 9.8% [30]. The chamois is one of the most important game species in Slovenia, with a population of over 10,000 individuals [53]. In our previous study, infectious diseases (82.2%) were identified as the primary cause of mortality in chamois, followed by non-infectious conditions (11.8%). Among all deaths, parasitic infections accounted for 70.3%, trauma for 9.7%, and bacterial infections for 9.3% [31]. In addition to our observations, European studies consistently show that gastrointestinal helminths are common, widespread, and ecologically significant in wild ungulates, particularly roe deer, mouflon, and fallow deer. A recent meta-analysis of multiple European cervid populations reported a high prevalence of gastrointestinal nematodes, including *Haemonchus contortus*, and Trichostrongylus spp., in roe and fallow deer, with infection pressures influenced by habitat and host interactions [54]. Detailed necropsy-based investigations in central Italy further confirmed the widespread presence of intestinal helminths in roe deer, often involving multiple taxa per animal [55]. Long-term health surveillance data from Switzerland indicate that gastrointestinal and other internal parasites were regularly recorded in necropsied roe deer and were associated with inflammation of the gastrointestinal mucosa and, in some cases, with clinical disease or death, highlighting their real contribution to mortality in natural populations [41]. Epidemiological surveys in Spain also documented high prevalences of gastrointestinal nematodes in roe deer, with some regions showing an association between heavy infection burdens and clinical signs that could increase mortality risk [56,57]. These European findings support our observations that intestinal parasitism is not merely incidental but, under certain ecological or host-specific conditions, can contribute significantly to morbidity and death in wild ungulates.

Our findings indicate that multiple endoparasitism is common among Slovenian wild ruminants, which frequently harbour co-infections. These observations are consistent with studies on European wild ungulates, where polyparasitism has been widely documented in cervid populations and other free-ranging ruminants [41,58,59,60]. Long-term health surveillance of roe deer in Switzerland revealed persistent co-occurrence of gastrointestinal and pulmonary parasites over decades, highlighting that multi-parasite infections are a stable and characteristic feature of wild cervid populations [41]. Such co-infections are likely shaped by host ecology, social behaviour, and habitat overlap, with more gregarious ungulate species exhibiting greater parasite diversity and infection risk [61]. From an epidemiological perspective, these findings emphasise the importance of adopting a multi-parasite framework, particularly at the wildlife–livestock interface, where co-infected wild ruminants may act as reservoirs for generalist parasites affecting domestic animals [62]. Integrating long-term and cross-regional data, as demonstrated in Swiss monitoring programmes, provides valuable insight into the ecological and health implications of polyparasitism in natural systems [41]. Despite increasing recognition of polyparasitism in wildlife, many studies still rely on single-parasite approaches, potentially underestimating both the ecological complexity and epidemiological significance of parasite communities in wild ruminants [63,64].

Wild boar is the main large game species hunted in Europe, so we would expect a higher number of carcasses. Finding dead wild boar is challenging due to several ecological and practical factors. They are rarely detected in natural landscapes due to dense vegetation, scavenger activity, rapid decomposition, and variable accessibility, highlighting the need for targeted search strategies in wildlife disease surveillance [65,66,67]. The number of wild boar carcasses found is increasing in Slovenia due to the presence of ASF in Europe, systematic searches, and financial rewards for recovered carcasses. However, because of biosecurity measures, a systematic examination of the cause of death is not conducted; usually, only samples are taken to test for the presence of the ASF virus. A similar pattern is observed in red deer, as carcasses are seldom found. They rarely exhibit clinically apparent disease or disease-related deaths in natural environments, since many infections remain subclinical and mortality is mainly due to predation, starvation, hunting, or accidents [68,69,70]. The European brown hare population has declined significantly across Europe since the 1960s, primarily due to agricultural intensification, habitat loss, decreased food availability, and landscape homogenisation, and in the 1980s due to infectious diseases, particularly European brown hare syndrome (EBHS) [71,72,73]. We also observe a similar trend in Slovenia, where EBHS was the cause of death in most examined European brown hares. The higher number of hare carcasses examined in our study is due to hunters’ awareness of the threat to hares from EBHS and other diseases, such as tularemia, particularly in areas where hares are present. At the same time, the hare is an important game species and is also used as food. The situation is different for the red fox, whose numbers in the wild are higher than those of the European brown hare. However, for hunters, the species is less important as a trophy, and consequently fewer carcasses are collected, leaving more in the natural environment. A significant factor is the occurrence of sarcoptic mange, one of the most important diseases in red foxes in Europe [74,75,76], which compromises coat integrity and results in pelts unsuitable for fur harvesting [77,78]. Sarcoptic mange is recognised as a major threat to ibex populations, often causing substantial morbidity and mortality and disrupting the normal physiological condition of affected individuals [79,80,81]. Similar findings have been observed in our necropsy results. Canine distemper virus (CDV) is a widespread pathogen in wild mustelids, including beech martens and European polecat. Infected individuals often exhibit respiratory and neurological signs, with rapid progression to death [82,83]. CDV prevalence in free-ranging mustelids varies but poses a considerable conservation concern, especially when combined with other stressors or infections [84,85]. These findings underscore the importance of viral infections in small carnivores as contributors to wildlife mortality.

In Europe, large carnivores such as the Eurasian lynx, wolf, and brown bear predominantly face human-caused mortality [86,87]. Traffic accidents are a widespread and increasing cause of wildlife mortality across Europe, affecting both mammals and birds [88,89]. In Slovenia, wildlife–vehicle collisions are a significant cause of mortality for large and medium-sized mammals. A comprehensive study along the Slovenian highway network recorded over 2000 roadkill incidents involving target mammalian species, including foxes, roe deer, badgers, and brown hares, over a three-year period, demonstrating clear temporal patterns and collision hotspots [90]. In several wildlife species, firearm-related injuries were recorded during post-mortem examinations, indicating that gunshot was a contributing factor to mortality. Large mammals, including red deer, wild boar, brown bears, wolves, and lynx, frequently showed evidence of gunshot trauma at necropsy, consistent with European studies documenting both legal and illegal shooting as a significant source of mortality in ungulates and large carnivores [91,92]. Similarly, smaller carnivores such as foxes and martens occasionally presented with firearm-related lesions, and necropsy-based studies in stone martens and other mustelids confirm gunshot as a direct or contributing cause of death [93,94]. Firearm injuries have also been documented in other taxa through necropsy examinations, including birds, highlighting the broad taxonomic relevance of gunshot as a mortality factor [95]. These findings show that firearms contribute directly to observed mortality, and careful necropsy is essential to distinguish gunshot injuries from other causes such as traffic accidents, disease, or natural predation. In our study, gunshot injuries were consistently documented as a primary or secondary factor in post-mortem assessments, highlighting their significance across European wildlife populations.

Among birds, the most notable case is the death of a Eurasian eagle owl from electrocution. In Slovenia, the Eurasian eagle-owl, one of Europe’s largest owl species, faces significant threats from electrical infrastructure. Recent monitoring under Natura 2000 programmes estimates approximately 100–150 breeding pairs in the country, although numbers may vary regionally depending on habitat availability and long-term population trends [96,97]. These birds suffer unnatural mortality when perching on inadequately insulated medium-voltage power lines, as their large wingspans can easily bridge energised components, resulting in fatal electrocution [98,99]. In recent years, at least 26 confirmed electrocution deaths have been recorded over two years [97]. This threat, along with habitat disturbance and collisions with other infrastructure, is considered a key factor impacting the species locally. Conservation efforts, including retrofitting dangerous poles with insulation and implementing bird-safe designs, are underway and aim to substantially reduce mortality rates, thereby supporting population stability [97].

Small mammal and bird carcasses are particularly difficult to detect in natural ecosystems due to rapid removal and environmental concealment. Studies indicate that habitat complexity plays a critical role in carcass persistence, as dense vegetation or canopy cover can obscure remains from both scavengers and researchers [100,101,102]. Additionally, carcass size strongly influences detectability, with smaller carcasses, such as those of small mammals and birds, being removed more quickly than larger ones [103,104,105]. These factors together significantly limit the opportunity for researchers to observe small wildlife mortality in the field, complicating efforts to assess population dynamics, disease monitoring, and ecological impacts. In addition, population size and density can strongly influence the number of carcasses found and submitted for investigation [3]. Mortalities of species at high densities or in aggregations tend to be more visible and detectable during surveys, whereas sparse or cryptic populations are less likely to have carcasses located [3].

## 5. Conclusions

Long-term passive wildlife surveillance in Slovenia has provided critical insights into the causes of mortality and disease concurrency across multiple species. Our analyses show that parasitic and bacterial infections are the main contributors to mortality in key ungulate species, such as roe deer and chamois, reflecting both population abundance and targeted monitoring efforts. However, carcass detectability, scavenger activity, and reporting biases strongly influence which species and individuals are represented, particularly among small mammals, birds, and elusive large game. Despite these limitations, necropsy-based surveillance remains a reliable tool for identifying the presence or absence of specific diseases, understanding broad mortality patterns, and informing wildlife health management and disease prevention strategies. Continued collaboration among veterinary services, hunters, and wildlife management organisations is essential to maintain and improve the effectiveness of national surveillance systems and to ensure timely detection of emerging pathogens that may impact wildlife, domestic animals, and public health. Wildlife health surveillance in Europe remains heterogeneous, with some countries running comprehensive programmes while others depend on disease-specific monitoring or passive reporting. Effective surveillance requires collaboration among stakeholders, including hunters, local communities, and citizen scientists, as well as coordination through networks such as the EWDA. Strengthening standardisation, cross-border collaboration, and One Health approaches is essential to improve early detection and preparedness for emerging wildlife diseases.

## Figures and Tables

**Table 1:** Number of post-mortem examinations of various free-ranging wild mammal and avian species at the Veterinary Faculty as part of a national health surveillance programme in Slovenia over 30 years (1995–2025), with primary causes of mortality or disease shown as percentages.

Mammal Species	*n*	Main Pathological Cause of Death or Disease (%)	Avian Species	*n*	Main Pathological Cause of Death or Disease (%)
Roe deer (*Capreolus capreolus*)	989	Multiple endoparasitism (26); *Haemonchus contortus* (16) bacterial infection (*Trueperella pyogenes*, *Bibersteinia trehalose*, etc.) (16)	Pheasant (*Phasianus colchicus*)	84	Trauma (29); firearms (23); *Mycoplasma gallisepticum* (20)
Chamois (*Rupicapra rupicapra*)	392	*Sarcoptes scabiei* (47); multiple endoparasitism (16) Protostrongylidae (11)	Blue heron (*Ardea herodias*)	10	Traffic accidents (60); cachexia (40)
Brown hare (*Lepus europaeus*)	201	EBHS (38); MAP (19) *Brucella suis* (13)	Common buzzard (*Buteo buteo*)	8	Traffic accidents (50); firearms (25); decomposed carcass (25)
Red deer (*Cervus elaphus*)	150	Traffic accidents (38); firearms (29); trauma (22)	Eagle (*Aquila chrysaetos*)	6	Starvation (50) Cachexia (25); decomposed carcass (25)
Fox (*Vulpes vulpes*)	128	Traffic accidents (41); *Sarcoptes scabiei* (24) canine distemper (19)	Grey partridge (*Perdix perdix*)	6	Predation (50); decomposed carcass (50)
Wild boar *(Sus scrofa*)	91	Firearms (31); traffic accidents (27); bacterial infection (*Staphylococcus aureus*, *Corynebacterium ulcerans*, etc.) (21)	Mallard (*Anas platyrhynchos*)	5	Firearms (60); *Sarcocystis rileyi* (40)
Fallow deer (*Cervus dama*)	79	Multiple endoparasitism (23); traffic accidents (19) metabolic disorder (12)	Swan (*Cygnus olor*)	5	Traffic accidents (80); Firearms (20)
Beech marten (*Martes foina*)	61	Traffic accidents 27); canine distemper (16) firearms (16)	Eurasian woodcock (*Scolopax rusticola*)	4	Cachexia (100)
Badger (*Meles meles*)	57	Traffic accidents (37); canine distemper (21) firearms (18)	Western capercaillie (*Tetrao urogallus*)	2	Coli sepsis (50); predation (50)
Brown bear (*Ursus arctos*)	44	Traffic accidents (43); firearms (39); undetermined cause (14)	Common kestrel (*Falco tinnunculus*)	2	Firearms (100)
European mouflon (*Ovis amon musimon*)	44	*Sarcoptes scabiei* (34); multiple endoparasitism (13) Protostrongylidae (8)	Seagull (*Larus canus*)	2	Firearms (100)
Wolf (*Canis lupus*)	40	Firearms (46); Traffic accidents (43) undetermined cause (6)	Tawny owl (*Strix aluco*)	1	Decomposed carcass (100)
Linx (*Lynx lynx*)	23	Firearms (52); traffic accidents (26); starvation (13)	Vulture (*Gyps fulvus*)	1	Cachexia (100)
Alpine ibex (*Capra ibex*)	19	*Sarcoptes scabiei* (84); multiple endoparasitism (10) decomposed carcass (6)	Crow (*Corvus cornix*)	1	Unknown (100)
European wildcat (*Felis silvestris*)	13	Traffic accidents (46); mixed bacterial flora (24); decomposed carcass (30)	Northern goshawk (*Accipiter gentilis*)	1	Predation (100)
Otter (*Lutra lutra*)	9	Traffic accidents (55); dog predation (45)	Black grouse (*Lyrurus tetrix*)	1	Decomposed carcass (100)
Golden jackal (*Canis aureus*)	8	Traffic accidents (100)	Turtle dove (*Streptopelia turtur*)	1	Predation (100)
Nutria (*Myocastor coypus*)	6	Traffic accidents (55); dog predation (45)	Yaj (*Garrulus glandarius*)	1	Trauma (100)
European polecat (*Mustela putorius*)	5	Traffic accidents (60); canine distemper (40)	Eurasian eagle-owl (*Bubo bubo*)	1	Electricity (100)
Pine marten (*Martes martes*)	5	Traffic accidents (60); canine distemper (40)			
Red squirrel (*Sciurus vulgaris*)	4	Traffic accidents (75); decomposed carcass (25)			
European beaver (*Castor fiber*)	2	Traffic accidents (50); dog predation (50)			

*n*—number of necropsies over 30 years; EBHS—European brown hare syndrome; MAP—Mycobacterium avium subsp. paratuberculosis.

## Data Availability

The raw data supporting the conclusions of this article will be made available by the authors on request.

## References

[1] Skoberne P., Tucker G. (2023). Slovenia. Nature Conservation in Europe.

[2] MESP-EARS (2001). Biological and Landscape Diversity in Slovenia.

[3] Mörner T., Obendorf D.L., Artois M., Woodford M.H. (2002). Surveillance and monitoring of wildlife diseases. Rev. Sci. Tech..

[4] Holmes J.P., Duff J.P., Barlow A., Everest D., Man C., Smith F., Twomey F. (2019). 20 years of national wildlife disease surveillance. Vet. Rec..

[5] Cardoso B., García-Bocanegra I., Acevedo P., Cáceres G., Alves P.C., Gortázar C. (2022). Stepping up from wildlife disease surveillance to integrated wildlife monitoring in Europe. Res. Vet. Sci..

[6] Daszak P., Cunningham A.A., Hyatt A.D. (2000). Emerging infectious diseases of wildlife—Threats to biodiversity and human health. Science.

[7] Karesh W.B., Dobson A., Lloyd-Smith J.O., Lubroth J., Dixon M.A., Bennett M., Aldrich S., Harrington T., Formenty P., Loh E.H. (2012). Ecology of zoonoses: Natural and unnatural histories. Lancet.

[8] Kuiken T., Ryser-Degiorgis M.P., Gavier-Widén D., Gortázar C. (2011). Establishing a European network for wildlife health surveillance. Rev. Sci. Tech. Int. Off. Epizoot..

[9] Ryser-Degiorgis M.P., Pewsner M., Angst C. (2015). Joining the dots—Understanding the complex interplay between the values we place on wildlife, biodiversity conservation, human and animal health: A review. Schweiz. Arch. Tierheilkd..

[10] Delgado M., Ferrari N., Fanelli A., Muset S., Thompson L., Sleeman J.M., White C.L., Walsh D., Wannous C., Tizzani P. (2023). Wildlife health surveillance: Gaps, needs and opportunities. Rev. Sci. Tech..

[11] Zinsstag J., Schelling E., Waltner-Toews D., Tanner M. (2011). From “one medicine” to “one health” and systemic approaches to health and well-being. Prev. Vet. Med..

[12] Geering W.A., Roeder P.L., Obi T.U. (1999). Manual on the Preparation of National Animal Disease Emergency Preparedness Plans.

[13] Mörner T., Beasley V., Norrgren L., Levengood J. (2012). Monitoring for diseases in wildlife populations. Ecology and Animal Health, Ecosystem Health and Sustainable Agriculture 2.

[14] Gervasi V., Marcon A., Bellini S., Guberti V. (2020). Passive surveilance in the detection of African swine fever in wild boar. Vet. Sci..

[15] Murray J., Cohen A. (2017). Infectious Disease Surveillance. International Encyclopedia of Public Health.

[16] Linnell J.D.C., Aanes R., Andersen R. (1995). Who killed Bambi? The role of predation in the neonatal mortality of temperate ungulates. Wildl. Biol..

[17] Küker S., Faverjon C., Furrer L., Berezowski J., Posthaus H., Rinaldi F., Vial F. (2018). The value of necropsy reports for animal health surveillance. BMC Vet. Res..

[18] Zanet S., Benatti F., Poncina M., Pasetto C., Chiari M., Sorrenti M., Ferroglio E. (2024). The Role of Hunters in Wildlife Health Research and Monitoring: Their Contribution as Citizen Scientists in Italy. Animals.

[19] Urner N., Mõtus K., Nurmoja I., Schulz J., Sauter-Louis C., Staubach C., Conraths F.J., Schulz K. (2020). Hunters’ Acceptance of Measures against African Swine Fever in Wild Boar in Estonia. Prev. Vet. Med..

[20] Redding D.W., Tiedt S., Lo Lacono G., Bett B., Jones K.E. (2017). Spatial, seasonal and climatic predictive models of Rift valley fever disease across Africa. Phil. Trans. R. Soc. B.

[21] Abrantes A.C., Vieira-Pinto M. (2023). 15 years overview of European zoonotic surveys in wild boar and red deer: A systematic review. One Health.

[22] Martin C., Pastoret P.P., Brochier B., Humblet M.F., Saegerman C. (2011). A survey of the transmission of infectious diseases/infections between wild and domestic ungulates in Europe. Vet. Res..

[23] Gortazar C., Ferroglio E., Höfle U., Frölich K., Vicente J. (2007). Diseases shared between wildlife and livestock: A European perspective. Eur. J. Wildl. Res..

[24] Yon L., Duff J.P., Ågren E.O., Erdélyi K., Ferroglio E., Godfroid J., Hars J., Hestvik G., Horton D., Kuiken T. (2019). Recent changes in infectious diseases in European wildlife. J. Wildl. Dis..

[25] Vada R., Zanet S., Ferroglio E. (2022). Fifty Years of Wildlife Diseases in Europe: A Citation Database Meta-Analysis. Vet. Sci..

[26] de Cock M.P., Baede V.O., Wijburg S.R., Burt S.A., van Tiel R.F., Wiskerke K.K., van der Post J.R., van der Poel W.H., Sprong H., Maas M. (2024). WILDbase: Towards a common database to improve wildlife disease surveillance in Europe. Eurosurveillance.

[27] Heiderich E., Keller S., Pewsner M., Origgi F.C., Zürcher-Giovannini S., Borel S., Marti I., Scherrer P., Pisano S.R.R., Friker B. (2024). Analysis of a European general wildlife health surveillance program: Chances, challenges and recommendations. PLoS ONE.

[28] Lawson B., Neimanis A., Lavazza A., López-Olvera J.R., Tavernier P., Billinis C., Duff J.P., Mladenov D.T., Rijks J.M., Savić S. (2021). How to Start Up a National Wildlife Health Surveillance Programme. Animals.

[29] Ryser-Degiorgis M.P. (2013). Wildlife health investigations: Needs, challenges and recommendations. BMC Vet. Res..

[30] Žele Vengušt D., Kuhar U., Jerina K., Vengušt G. (2021). Twenty Years of Passive Disease Surveillance of Roe Deer (*Capreolus capreolus*) in Slovenia. Animals.

[31] Vengušt G., Kuhar U., Jerina K., Švara T., Gombač M., Bandelj P., Vengušt D. (2022). Passive Disease Surveillance of Alpine Chamois (*Rupicapra r. rupicapra*) in Slovenia between 2000 and 2020. Animals.

[32] McAloose D., Colegrove K.M., Newton A.L., Terio K.A., McAloose D., Leger J. (2018). Wildlife necropsy. Pathology of Wildlife and Zoo Animals.

[33] Soulsby E.J.L. (1982). Helminths, Arthropods and Protozoa of Domesticated Animals.

[34] Taylor M.A., Coop R.L., Wall R.L. (2016). Veterinary Parasitology.

[35] Ecker J., Rommel M., Ecker J., Kurtzer W., Körting E., Schnieder T. (2000). Helminthologische Methoden. Veterinärmedizinische Parasitologie.

[36] Deplazes P., Eckert J., Samson-Himmelstjerna G., Zahner H. (2012). Lehrbuch der Parasitologie für die Tiermedizin.

[37] Boch J., Supperer R. (2006). Veterinärmedizinische Parasitologie.

[38] Woolhouse M.E. (2002). Population biology of emerging and re-emerging pathogens. Trends Microbiol..

[39] Jones K.E., Patel N.G., Levy M.A., Storeygard A., Balk D., Gittleman J.L., Daszak P. (2008). Global trends in emerging infectious diseases. Nature.

[40] Allen T., Murray K.A., Zambrana-Torrelio C., Morse S.S., Rondinini C., Di Marco M., Breit N., Olival K.J., Daszak P. (2017). Global hotspots and correlates of emerging zoonotic diseases. Nat. Commun..

[41] Pewsner M., Origgi F.C., Frey J., Ryser-Degiorgis M.P. (2017). Assessing Fifty Years of General Health Surveillance of Roe Deer in Switzerland: A Retrospective Analysis of Necropsy Reports. PLoS ONE.

[42] Cunningham A.A., Daszak P., Wood J.L.N. (2017). One Health, emerging infectious diseases and wildlife: Two decades of progress?. Philos. Trans. R. Soc. Lond. B Biol. Sci..

[43] Schmeller D.S., Courchamp F., Killeen G. (2020). Biodiversity loss, emerging pathogens and human health risks. Biodivers. Conserv..

[44] Barroso P., Relimpio D., Zearra J.A., Cerón J.J., Palencia P., Cardoso B., Ferreras E., Escobar M., Cáceres G., López-Olvera J.R. (2023). Using integrated wildlife monitoring to prevent future pandemics through one health approach. One Health.

[45] World Organisation for Animal Health (2015). Guidelines for Wildlife Disease Surveillance: An Overview.

[46] Stallknecht D.E. (2007). Impediments to wildlife disease surveillance, research, and diagnostics. Curr. Top. Microbiol. Immunol..

[47] Duncan C., Backus L., Lynn T., Powers B., Salman M. (2008). Passive, opportunistic wildlife disease surveillance in the Rocky Mountain Region, USA. Transbound. Emerg. Dis..

[48] Akdesir E., Origgi F.C., Wimmershoff J., Frey J., Frey C.F., Ryser-Degiorgis M.P. (2018). Causes of mortality and morbidity in free-ranging mustelids in Switzerland: Necropsy data from over 50 years of general health surveillance. BMC Vet. Res..

[49] Kuiken T., Leighton F.A., Fouchier R.A., LeDuc J.W., Peiris J.S., Schudel A., Stöhr K., Osterhaus A.D. (2005). Public health. Pathogen surveillance in animals. Science.

[50] Lamarque F., Barrat J., Hatier C., Artois M. (1999). Causes of mortality in roe deer (*Capreolus capreolus*) diagnosed by an epidemiological surveillance network in France. Gibier Faune Sauvage Fr..

[51] Aguirre A.A., Bröjer C., Mörner T. (1999). Descriptive epidemiology of roe deer mortality in Sweden. J. Wildl. Dis..

[52] Jerina K., Stergar M., Jelenko I., Pokorny B. (2010). Spatial Distibution, Fitness, and Population Dynamics of Ungulates in Slovenia: Studies on the Effects of Spatially Explicite Habitat and Species-Specific Factors and Predicting Future Trends.

[53] Adamič M., Jerina K., Apollonio M., Andersen R., Putman R. (2010). Ungulates and their management in Slovenia. European Ungulates and Their Management in the 21st Century.

[54] Brown T.L., Morgan E.R. (2024). Helminth Prevalence in European Deer with a Focus on Abomasal Nematodes and the Influence of Livestock Pasture Contact: A Meta-Analysis. Pathogens.

[55] Macchioni F., Vallone F., Lenzi C., Monni G., Matiacic A., Cecchi F., Romeo G. (2023). Helminth fauna in roe deer (*Capreolus capreolus* Linnaeus, 1758) in the province of Grosseto (central Italy). Helminthologia.

[56] González S., Del Rio M.L., Díez-Baños N., Martínez A., Hidalgo M.D.R. (2023). Contribution to the Knowledge of Gastrointestinal Nematodes in Roe Deer (*Capreolus capreolus*) from the Province of Leon, Spain: An epidemiological and molecular study. Animals.

[57] Pato F.J., Vázquez L., Díez-Baños N., López C., Sánchez-Andrade R., Fernández G., Díez-Baños P., Panadero R., Díaz P., Morrondo P. (2013). Gastrointestinal nematode infections in roe deer (*Capreolus capreolus*) from the NW of the Iberian Peninsula: Assessment of some risk factors. Vet. Parasitol..

[58] Davidson R.K., Kutz S.J., Madslien K., Hoberg E., Handeland K. (2014). Gastrointestinal parasites in an isolated Norwegian population of wild red deer (*Cervus elaphus*). Acta Vet. Scand..

[59] Carrau T., Martínez-Carrasco C., Garijo M.M., Alonso F., Vizcaíno L.L., Herrera-Russert J., Tizzani P., Ruiz de Ybáñez R. (2021). Epidemiological approach to nematode polyparasitism occurring in a sympatric wild ruminant multi-host scenario. J. Helminthol..

[60] Beaumelle C., Redman E.M., de Rijke J., Wit J., Benabed S., Debias F., Duhayer J., Pardonnet S., Poirel M.T., Capron G. (2021). Metabarcoding in two isolated populations of wild roe deer (*Capreolus capreolus*) reveals variation in gastrointestinal nematode community composition between regions and among age classes. Parasites Vectors.

[61] Ezenwa V.O. (2004). Interactions among host diet, nutritional status and gastrointestinal parasite infection in wild bovids. Int. J. Parasitol..

[62] Walker J.G., Morgan E.R. (2014). Generalists at the interface: Nematode transmission between wild and domestic ungulates. Int. J. Parasitol. Parasites Wildl..

[63] Telfer S., Lambin X., Birtles R., Beldomenico P., Burthe S., Paterson S., Begon M. (2010). Species interactions in a parasite community drive infection risk in a wildlife population. Science.

[64] Vaumourin E., Vourc’h G., Gasqui P., Vayssier-Taussat M. (2015). The importance of multiparasitism: Examining the consequences of co-infections for human and animal health. Parasites Vectors.

[65] Rogoll L., Schulz K., Staubach C., Oļševskis E., Seržants M., Lamberga K., Conraths F.J., Sauter-Louis C. (2024). Identification of predilection sites for wild boar carcass search based on spatial analysis of Latvian ASF surveillance data. Sci. Rep..

[66] Rietz J., van Beeck Calkoen S.T.S., Ferry N., Schlüter J., Wehner H., Schindlatz K.H., Lackner T., von Hoermann C., Conraths F.J., Müller J. (2023). Drone-Based Thermal Imaging in the Detection of Wildlife Carcasses and Disease Management. Transbound. Emerg. Dis..

[67] Rietz J., Ischebeck S., Conraths F.J., Probst C., Zedrosser A., Fiderer C., Reckel F., von Hoermann C., Müller J., Heurich M. (2024). Scavenger-induced scattering of wild boar carcasses over large distances and its implications for disease management. J. Environ. Manag..

[68] Kamler J.F., Jędrzejewski W., Jędrzejewska B. (2007). Survival and Cause-specific Mortality of Red Deer *Cervus elaphus* in Białowieża National Park, Poland. Wildl. Biol..

[69] Rossi S., Balenghien T., Viarouge C., Faure E., Zanella G., Sailleau C., Mathieu B., Delécolle J.C., Ninio C., Garros C. (2019). Red deer (*Cervus elaphus*) Did Not Play the Role of Maintenance Host for Bluetongue Virus in France: The Burden of Proof by Long-Term Wildlife Monitoring and Culicoides Snapshots. Viruses.

[70] Borowik T., Wawrzyniak P., Jedrzejewska B. (2016). Red deer (*Cervus elaphus*) fertility and survival of young in a low-density population subject to predation and hunting. J. Mammal..

[71] Pavliska P.L., Riegert J., Gril S., Šalek M. (2018). The effect of landscape heterogeneity on population density and habitat preferences of the European hare (*Lepus europaeus*) in contrasting farmlands. Mamm. Biol..

[72] Schmidt N.M., Asferg T., Forchhammer M.C. (2004). Long-term patterns in European brown hare population dynamics in Denmark: Effects of agriculture, predation and climate. BMC Ecol..

[73] Wirblich C., Meyers G., Ohlinger V.F., Capucci L., Eskens U., Haas B., Thiel H.J. (1994). European brown hare syndrome virus: Relationship to rabbit hemorrhagic disease virus and other caliciviruses. J. Virol..

[74] Willebrand T., Samelius G., Odden M., Englund J. (2022). Declining survival rates of red foxes *Vulpes vulpes* during the first outbreak of sarcoptic mange in Sweden. Wildl. Biol..

[75] Perrucci S., Verin R., Mancianti F., Poli A. (2016). Sarcoptic mange and other ectoparasitic infections in a red fox (*Vulpes vulpes*) population from central Italy. Parasite Epidemiol. Control.

[76] Pisano S.R.R., Zimmermann F., Rossi L., Capt S., Akdesir E., Bürki R., Kunz F., Origgi F.C., Ryser-Degiorgis M.P. (2019). Spatiotemporal spread of sarcoptic mange in the red fox (*Vulpes vulpes*) in Switzerland over more than 60 years: Lessons learnt from comparative analysis of multiple surveillance tools. Parasites Vectors.

[77] Nimmervoll H., Hoby S., Robert N., Lommano E., Welle M., Ryser-Degiorgis M.P. (2013). Pathology of sarcoptic mange in red foxes (*Vulpes vulpes*): Macroscopic and histologic characterization of three disease stages. J. Wildl. Dis..

[78] Barroso P., Palencia P. (2024). Camera traps reveal a high prevalence of sarcoptic mange in red foxes from northern Spain. Res. Vet. Sci..

[79] Pérez J.M., Granados J.E., Espinosa J., Ráez-Bravo A., López-Olvera J.R., Rossi L., Meneguz P.G., Angelone S., Fandos P., Soriguer R.C. (2021). Biology and management of sarcoptic mange in wild Caprinae populations. Mamm. Rev..

[80] Rossi L., Tizzani P., Rambozzi L., Moroni B., Meneguz P.G. (2019). Sanitary Emergencies at the Wild/Domestic Caprines Interface in Europe. Animals.

[81] Unterköfler M.S., Schausberger M., Deutz A., Gressmann G., Kübber-Heiss A., Ferroglio E., Joachim A. (2023). Sarcoptic mange in wild ungulates in the European Alps—A systematic review. Int. J. Parasitol. Parasites Wildl..

[82] Ke G.M., Ho C.H., Chiang M.J., Sanno-Duanda B., Chung C.S., Lin M.Y., Shi Y.Y., Yang M.H., Tyan Y.C., Liao P.C. (2015). Phylodynamic analysis of the canine distemper virus hemagglutinin gene. BMC Vet. Res..

[83] Alfano F., Lucibelli M.G., D’Alessio N., Auriemma C., Rea S., Sgroi G., Lucente M.S., Pellegrini F., Diakoudi G., De Carlo E. (2025). Detection of canine distemper virus in wildlife in Italy (2022–2024). Front. Vet. Sci..

[84] Lanszki Z., Lanszki J., Tóth G.E., Cserkész T., Csorba G., Görföl T., Csathó A.I., Jakab F., Kemenesi G. (2022). Detection and sequence analysis of *Canine morbillivirus* in multiple species of the Mustelidae family. BMC Vet. Res..

[85] Kličková E., Černíková L., Dumondin A., Bártová E., Budíková M., Sedlák K. (2022). Canine Distemper Virus in Wild Carnivore Populations from the Czech Republic (2012–2020): Occurrence, Geographical Distribution, and Phylogenetic Analysis. Life.

[86] Balseiro A., Herrero-García G., García Marín J.F., Balsera R., Monasterio J.M., Cubero D., de Pedro G., Oleaga Á., García-Rodríguez A., Espinoza I. (2024). New threats in the recovery of large carnivores inhabiting human-modified landscapes: The case of the Cantabrian brown bear (*Ursus arctos*). Vet. Res..

[87] Premier J., Bastianelli M.L., Oeser J., Anders O., Andren H., Aronsson M., Bagrade G., Belotti E., Breitenmoser-Würsten C., Bufka L. (2025). Survival of Eurasian lynx in the human-dominated landscape of Europe. Conserv. Biol..

[88] Grilo C., Koroleva E., Andrašik R., Bil M., Gonzales-Suarez M. (2020). Roadkill risk and population vulnerability in European birds and mammals. Front. Ecol. Environ..

[89] Bíl M., Balčiauskas L., Bílová M., Cellina S., Favilli F., Gačić D., Guinard E., Heurich M., Ivanova N., Junghardt J. (2025). Wildlife-vehicle collision liability in Europe: A review of existing approaches and their implications. J. Environ. Manag..

[90] Petkovšek S.S., Kotnik K., Breznik K., Pokorny B. (2025). Wildlife mortality on the Slovenian highways: Monthly patterns, identification of hotspots and effectiveness of acoustic deterrents. Urban Ecosyst..

[91] Chapron G., Kaczensky P., Linnell J.D., von Arx M., Huber D., Andrén H., López-Bao J.V., Adamec M., Álvares F., Anders O. (2014). Recovery of large carnivores in Europe’s modern human-dominated landscapes. Science.

[92] Milner J.M., Nilsen E.B., Andreassen H.P. (2007). Demographic side effects of selective hunting in ungulates and carnivores. Conserv. Biol..

[93] Balboni A., Savini F., Scagliarini A., Berti E., Naldi M., Urbani L., Fontana M.C., Carra E., Gibelli L.R.M., Gobbo F. (2021). Natural distemper infection in stone martens (*Martes foina*): From infection to neutralizing antibodies. Res. Vet. Sci..

[94] Pavlačik L., Celer V., Koubek P., Literak I. (2007). Prevalence of canine distemper virus in wild mustelids in the Czech Republic and a case of canine distemper in young stone martens. Vet. Med..

[95] Pankowski F., Bogiel G., Paśko S., Rzepiński F., Misiewicz J., Staszak A., Bonecka J., Dzierzęcka M., Bartyzel B.J. (2018). Fatal gunshot injuries in the common buzzard *Buteo buteo* L. 1758—Imaging and ballistic findings. Forensic Sci. Med. Pathol..

[96] Denac K., Božič L., Blažič B., Bordjan D., Koce U., Mihelič T., Očko U. Monitoring of Selected Target Bird Species Populations in Natura 2000 Sites in 2025. https://www.google.com/url?sa=t&source=web&rct=j&opi=89978449&url=https://ptice.si/wp-content/uploads/2025/11/Porocilo_monitoring_ptice_2025.pdf&ved=2ahUKEwjgvNj5rNuTAxWCnP0HHd81EnUQFnoECBoQAQ&usg=AOvVaw1dkQUP_Qsej2xO5LjjugIf.

[97] Life for Lifelines Eagle Owl (*Bubo bubo*). https://lifeforlifelines.ptice.si/en/vrste/velika-uharica-bubo-bubo/.

[98] Sergio F., Marchesi L., Pedrini P., Ferrer M., Penteriani V. (2004). Electrocution alters the distribution and density of a top predator, the eagle owl *Bubo bubo*. J. Appl. Ecol..

[99] Nygård T., Jacobsen K.O., Gjershaug J.O. (2023). Home-range, movements and use of powerline poles of Eagle-Owls (*Bubo bubo*) at an island population in northern Norway. Ornis Fenn..

[100] Daza R.R., Acebes P., Olea P.P. (2025). Farmland abandonment and season drive scavenging dynamics in livestock-rewilded landscapes. Ecol. Appl..

[101] Moleon M., Sanchez-Zapata J.A., Margalida A., Carrete M., Owen-Smith N., Donazar J.A. (2014). Humand snd scavengers: The evolution of interaction and ecosystem services. Bioscience.

[102] Borner L., Duriez O., Besnard A., Robert A., Carrere V., Jiguet F. (2017). Bird collision with power lines: Estimating carcass persistence and detection associated with ground search surveys. Ecosphere.

[103] O’Bryan C.J., Holden M.H., Watson J.E.M. (2019). The mesoscavenger release hypothesis and implications for ecosystem and human well-being. Ecol. Lett..

[104] Antworth R.L., Pike D.A., Stevens E.E. Hit and run: Effects of Scavenging on estimates of roadkolled vertebrates. Southeast. Nat. 4.

[105] Henry D.A.W., Collinson-Jonker W.J., Davies-Mostert H.T., Nicholson S.K., Roxburgh L., Parker D.M. (2021). Optimising the cost of roadkill surveys based on an analysis of carcass persistence. J. Environ. Manag..

